# How School and Home Contexts Impact the School Adjustment of Adolescents from Different Ethnic and SES Backgrounds During COVID-19 School Closures

**DOI:** 10.1007/s10964-023-01772-z

**Published:** 2023-04-21

**Authors:** Jessie Hillekens, Gülseli Baysu, Karen Phalet

**Affiliations:** 1 Department of Developmental Psychology, Tilburg University, Prof. Cobbenhagenlaan 225, 5037 DB Tilburg, the Netherlands; 2 Center for Social and Cultural Psychology, University of Leuven, Tiensestraat 102, 3000 Leuven, Belgium; 3 School of Psychology, Queen’s University Belfast, 2017 University Road, BT7 1NN Belfast, Northern Ireland UK

**Keywords:** COVID-19, School closures, School adjustment, Ethnic minority adolescents, Lower SES adolescents

## Abstract

Many schools worldwide closed to contain the spread of the COVID-19 virus. However, the consequences of school closures for the school adjustment of adolescents from different ethnic and SES backgrounds remain unclear. This study examined how school adjustment changed before, during, and after school closure across adolescents from different ethnic and SES backgrounds; and which factors in home and school contexts served as resources. Early adolescents (*N* = 124, *M*_age_ = 12.86, 58.8% boys) from different ethnic and SES backgrounds were repeatedly assessed 1 week before (March 2020), during (June 2020), and 1 year after (February 2021) the first school closure in Belgium. The results revealed that school closure augmented ethnicity- and SES-based inequalities in school adjustment. Moreover, factors in the school context—and not the home context—served as resources. Specifically, the quality of online instruction and teacher-pupil relationships buffered against reduced school adjustment during school closure, particularly among youth from ethnic minority and lower SES backgrounds. The findings corroborate unequal school adjustment consequences of school closures, but also highlight the role of teachers to buffer against them. The study design, hypotheses, and analyses were preregistered in the following link: https://osf.io/6ygcu/?view_only=c77cfb46028447bdb7844cd2c76237aa.

## Introduction

The COVID-19 pandemic has disrupted the lives of many, including those of adolescents. Governments across the world took far-reaching measures to contain the spread of the virus. As schools were considered a potential hotspot for virus transfer, many adolescents and their teachers were confronted with the closure of schools from one day to the next. While the beneficial effect of school closure on virus transmission is still debated (Koirala et al. 2021), both cross-sectional and longitudinal evidence suggests that school closures predict worse well-being among adolescents (Branje and Sheffield Morris, 2021 for a review). There are indications that containment measures generally affected individuals from ethnic minority and lower SES backgrounds more severely than those from ethnic majority and higher SES backgrounds. For example, they were more often key workers with a high risk to get infected. They were also more likely to lose their jobs during the pandemic with severe implications for the family income (Bambra et al. 2021). However, it remains unclear whether school closures differentially affected the school adjustment of adolescents from different ethnic and SES backgrounds; and what factors in school and home contexts can be a resource for sustaining adolescents’ adjustment in these challenging times. Addressing this gap, this preregistered study (https://osf.io/6ygcu/?view_only=c77cfb46028447bdb7844cd2c76237aa) sought to examine the impact of school closures on the school adjustment of adolescents from different ethnic and SES backgrounds. To this end, it uses data that was collected 1 week before (March 2020), during (June 2020), and 1 year after (February 2021) the first school closure in Belgium. Additionally, it asked how certain aspects of school and home contexts influence school adjustment during and after school closure, and whether these aspects vary across adolescents from different ethnic and SES backgrounds.

### Changes in School Adjustment Following School Closures

The outbreak of the COVID-19 virus and the following containment measures had a major impact on the daily lives of adolescents. Although adolescents were less likely to become seriously ill from the virus themselves, they were worried about the health of their families and loved ones (Achterberg et al. 2021). Some adolescents noticed their parents going to work as key workers, being on the frontline of the pandemic (Bambra et al. 2021). Others were worried about the family income when their parents lost their jobs (Achterberg et al. 2021). Besides worries about the virus and its consequences, adolescents were also confronted with several containment measures such as social distancing or the closure of shops. One of the most disruptive containment measures for adolescents was the closure of schools. School closures limited their opportunities to interact with their peers as a core socialization context (Rubin et al. 2008) and disrupted their daily routines immensely. School and home were previously separate social contexts, but they became increasingly intertwined during school closures. Adolescents were forced to spend entire days at home in the presence of their families while working on school tasks. Consequently, they felt lonely and isolated due to a lack of opportunity to meet with friends and peers (Romm et al. 2021); and they worried about the consequences of school closures for their school grades and achievement (Achterberg et al. 2021).

Arguably, adolescents’ school adjustment might also have been impacted by the pandemic and its consequences. School adjustment refers to how comfortable adolescents feel in school, how engaged they are with school tasks as well as their academic achievement (Demirtaş-Zorbaza and Ergeneb, 2020). It therefore comprises of several subdimensions, such as school belonging, school engagement, and academic self-esteem. According to a dynamic systems theory of development (Thelen and Smith, 2006), development can be seen as a system that comprises of interactions between different social actors. Adolescents thus constantly develop in relation to significant others in their environment (and also impact them and vice versa). Importantly, containment measures changed adolescents’ daily social interactions: Adolescents had less opportunities to meet with peers, teachers, and extended family, whereas they spent more time with their parents and siblings at home. Arguably, these changes in social interactions might have led to changes in developmental outcomes—including school adjustment.

Previous longitudinal evidence suggests that containment measures—like school closures—indeed negatively impacted adolescents’ school adjustment. Adolescents reported feeling isolated because they were not able to see their friends, peers, and teachers during online tutoring; and this in turn predicted worse school adjustment (Romm et al. 2021). Similarly, a recent daily dairy study conducted during lockdown showed that adolescents were less motivated and less engaged to do schoolwork on days that they received online education compared to the days that they could attend school physically (Klootwijk et al. 2021). Finally, most older children and adolescents reported decreased school engagement, increased school burnout (Salmela-Aro et al. 2021), and decreased school belonging (Maiya et al. 2021) during the lockdown compared to a year before. These studies therefore provide evidence of decreased school adjustment during school closures from 1 year before to during the pandemic. However, 1 year is a long time in adolescence during which other factors including developmental processes might have caused these changes in their school adjustment. Thus, it remains unclear whether and how school adjustment changed from shortly before to during school closures and how school adjustment evolved *after* school closures when schools reopened.

### The Role of School and Home Contexts

Although all adolescents were confronted with the COVID-19 pandemic and its consequences, some might be harder affected than others. Specifically, adolescents have different school and home contexts that likely impact changes in their school adjustment differently. In line with a dynamic systems theory of development (Thelen and Smith, 2006), different social dynamics and environments lead to different developmental pathways. Accordingly, adolescents’ different experiences in school and at home may lead to different school adjustment outcomes during the pandemic. For example, some adolescents might reside in more favorable social environments than others; and they might consequently have had an easier time sustaining their school adjustment during lockdown. Whereas previous research focused on changes in adolescents’ developmental outcomes during the COVID-19 pandemic (Branje and Sheffield Morris, 2021 for a review), less is known about the facets of school and home contexts that can sustain adolescents’ school adjustment during school closures.

Teachers may play a critical role in the school context in sustaining adolescents’ school adjustment during lockdown. Specifically, the quality of online instruction and the quality of teacher-pupil relationships seem relevant for distance learning and could act as protective factors. Quality of online instruction refers to a newly developed construct. It taps into how involved teachers are with their pupils during school closures, for example by correcting exercises or tasks or checking them together (Chrisman and Alnaim, 2021). The quality of teacher-pupil relationships refers to the experienced support and/or rejection from teachers. Although the quality of teacher-pupil relationships is essential for adolescents’ school adjustment regardless of the COVID-19 pandemic (see Baysu et al. 2021 for an empirical study; Roorda et al. 2011 for a review), it might be even more critical during school closures (Mastrotheodoros, 2020 for a similar argument). No previous studies examined the role of teachers in sustaining adolescents’ school adjustment during school closures.

The available resources at home and family support with homework might be critical facets in the home context. Existing longitudinal evidence confirms this idea. For instance, changes in academic self-esteem during school closures could be explained by adolescents’ available resources at home (Paizan et al. 2021). Moreover, parental support with homework was protective against decreased school belonging (Maiya et al. 2021) and academic self-esteem during school closures (Paizan et al. 2021). These studies provide first evidence of the importance of home contexts for adolescents’ school adjustment during school closures. However, siblings can also be an important source of support, particularly among youth from ethnic minority and lower SES backgrounds (Moguérou and Santelli, 2015). Although often neglected in developmental science, it is therefore important to ask adolescents about family support with homework rather than focusing solely on support from parents. Moreover, no research has been conducted shortly before and after school closures; or has examined the role of school contexts in sustaining adolescents’ school adjustment during school closures. More research is therefore needed to investigate which aspects of school and home contexts sustain adolescents’ school adjustment during school closures. Finally, there are indications that older adolescents (Engels et al. 2020), boys (Lietaert et al. 2015), and adolescents in vocational school tracks (Baysu et al. 2018) show lower school adjustment compared to their peers. It is therefore important to take these factors into account as potential confounding effects to more reliably show the protective role of home and school contexts during school closures.

### Differences across Adolescents from Different Ethnic and SES Backgrounds

The impact of the COVID-19 pandemic and its consequences has not only been experienced unequally, but it has also interacted with and augmented pre-existing inequalities (Bambra et al. 2021). For example, parents from ethnic minority and lower SES backgrounds were more likely to serve as key workers or lose their jobs during the pandemic (Bambra et al. 2021). Similarly, adolescents from ethnic minority and lower SES backgrounds also more often resided in less optimal circumstances during lockdown. In line with the integrative risk and resilience model for the adaptation of at-risk youth (Suárez-Orozco et al. 2018), adolescents from ethnic minority and lower SES backgrounds are confronted with multiple specific risk factors in their social environment that impact their developmental pathways. Consequently, their developmental pathways might differ from those of their peers from ethnic majority and higher SES backgrounds (Syed et al. 2018). It is therefore possible that adolescents from ethnic minority and lower SES backgrounds would show stronger decreases in school adjustment during school closures; and that these differences might be (partially) observed because they have less optimal school and home contexts compared to their peers.

Existing evidence, although limited, suggests that the COVID-19 pandemic may have had more detrimental consequences for the school adjustment of adolescents from ethnic minority and lower SES backgrounds. For example, cross-sectional studies demonstrated that pupils from ethnic minority (Bayrakdar and Guveli, 2020) and lower SES backgrounds spent less time on homework (Ariyo et al. 2022) and were less engaged during school closures (Easterbrook et al. 2022). Moreover, the few longitudinal studies conducted during lockdown showed a similar picture. For example, decreases in school belonging were steeper among youth from lower SES backgrounds (Maiya et al. 2021). Similarly, ethnic majority youth increased their academic self-esteem during lockdown compared to 1 year before, whereas ethnic minority youth did not show any differences (Paizan et al. 2021). Overall, this research suggests that school adjustment of pupils from ethnic minority and lower SES backgrounds might be harder affected during the pandemic.

Adolescents from ethnic minority and lower SES backgrounds might be affected harder due to their school and home contexts. This may play out in two ways. On the one hand, school and home contexts may be more consequential for school adjustment among youth from ethnic minority and lower SES backgrounds than those from ethnic majority and higher SES backgrounds. Adolescents from ethnic minority and lower SES backgrounds encounter multiple adversaries at a systemic level. Accordingly, positive relations in their immediate environment may (partially) buffer against potential negative consequences (Suárez-Orozco et al. 2018). This would technically be a moderation test. On the other hand, ethnicity or SES might shape adolescents’ experience of the home and school context during COVID-19 school closures, and in turn their school adjustment during and beyond this period. More specifically, school and home contexts of adolescents from ethnic minority and lower SES backgrounds might be less optimal than those of their peers during school closures. For instance, they might have less supportive social interactions or less help with homework. Consequently, these suboptimal circumstances might in turn translate into worse school adjustment (García Coll et al. 1996). This would technically be a mediation test.

Previous evidence supports both ideas. First, there is support for the moderation idea. For example, more resources at home and more parental support with homework were associated with better school adjustment during lockdown, but only among adolescents from lower SES backgrounds (Easterbrook et al. 2022). Similarly, how much distance learning was expected and how often homework was checked by teachers reduced school adjustment gaps between children from ethnic minority and lower SES backgrounds and their peers (Bayrakdar and Guveli, 2020). Second, there is support for the mediation idea. In the home context, adolescents from lower SES (Ariyo et al. 2021) and ethnic minority backgrounds (Paizan et al. 2021) had fewer resources to learn from home during lockdown. Similarly, adolescents from lower SES backgrounds received less parental support with homework during school closure (Paizan et al. 2021). In the school context, children from ethnic minority and lower SES backgrounds received lower quality of online instruction during school closures (González & Bonal, 2021); and research before the pandemic shows that ethnic minority youth had more negative relationships with their teachers (Baysu et al. 2021). These restricted opportunities in the home and school context thus point toward a cumulative disadvantage among youth from ethnic minority and lower SES backgrounds (González & Bonal, 2021). Still, most prior research on the COVID-19 pandemic did not distinguish between adolescents from different ethnic and SES backgrounds; and studies that made the distinction were mainly cross-sectional (see Maiya et al. 2021 and Paizan et al. 2021 for notable exceptions). More research is thus needed to investigate whether changes in school adjustment and potential protective facets in home and school contexts during the COVID-19 pandemic differ between adolescents from different ethnic and SES backgrounds.

## The Current Study

Although the COVID-19 pandemic and its consequences have disrupted the lives of many, few longitudinal studies have examined how they affected the school adjustment of adolescents from different ethnic and SES backgrounds. The aims of the present study were therefore threefold. First, this study examined adolescents’ school adjustment 1 week before, during, and 1 year after the first school closure in Belgium. It was expected that adolescents would show a decrease in school adjustment from before to during school closure (Hypothesis 1); and it was explored how school adjustment changed after a year when schools reopened. Second, this study asked how certain aspects of school and home contexts influence adolescents’ school adjustment during and after school closure. It was expected that adolescents in less (vs. more) positive school and home contexts would show decreased school adjustment during school closure (Hypothesis 2); and it was explored whether there would be any effects on school adjustment after a year. Third, this study asked whether and how these effects would be different for adolescents from different ethnic and SES backgrounds. It was expected that adolescents from ethnic minority and lower SES backgrounds would show worse school adjustment from before to during school closure than those from ethnic majority and higher SES backgrounds (Hypothesis 3.1); and it was explored how school adjustment after a year depended on adolescents’ ethnic and SES backgrounds. This study also investigated the role of adolescents’ ethnic and SES backgrounds in the impact of school and home contexts on school adjustment during and after school closure. It was expected that this would play out in two ways: 1) the effects of school and home contexts on school adjustment would be stronger for adolescents from ethnic minority and lower SES backgrounds (Hypothesis 3.2; moderation model); and 2) adolescents from ethnic minority and lower SES backgrounds would experience less positive school and home contexts and in turn worse school adjustment (Hypothesis 3.3; mediation model, not preregistered). Finally, the current study included age, gender, and school track as control variables.

## Method

### Participants

Adolescents (*N* = 124) in the first 2 years of a secondary school in Belgium participated in this study^1^. Adolescents were on average 12.86 years old at time one (SD = 0.91, range: 11–15 years), there were slightly more boys (58.8%) than girls (40.3%; 0.8% identified as non-binary). Approximately half of the adolescents attended vocational school tracks preparing pupils for the labor market (43.5 vs. 56.5% in academic or professional tracks preparing for higher education) and had lower educated parents (50.9% whose parents completed primary or secondary education vs. 49.1% whose parents completed higher education). Adolescents reported an average subjective SES of 7.84 (*SD* = 1.73, range: 2–10), with 17.1% reporting a subjective SES of 6 or lower. Adolescents’ ethnic backgrounds were based on self-reported parentage: Those with at least one (grand)parent born abroad were considered to be from ethnic minority backgrounds, in line with previous studies in Europe (e.g., Baysu et al. 2021). Adolescents from ethnic minority backgrounds (47.9 vs. 52.1% ethnic majority) were mostly second or later generations (69.6 vs. 30.4% first generation) and originated from 27 different countries across the world. Although the sample size was small, the design of the study made it impossible to collect additional data or conduct a priori power analyses. Data was collected already before school closure started and consequently all adolescents with available data before school closure were followed up during and after school closure. Post-hoc and sensitivity power analyses are criticized for its incorrect interpretation due to false assumptions on data characteristics (Dziak et al. 2021). Still, a sensitivity analysis was conducted in G*Power version 3.1.9.7. for the sake of transparency regarding statistical power. The main aim of the present study was to examine the predictive effects of home and school contexts on school adjustment during school closure. The primary interest was therefore the potential significance of the regression coefficients—which follow a *t*-test distribution. Therefore, the *t*-test family was chosen with ‘Linear multiple regression: Fixed model, single regression coefficient’ as statistical test with 7 predictors, an alpha of 0.05, and a power of 0.80. This analysis showed that the sample size was sufficient to detect a small to medium effect of 0.06. The study thus had sufficient power to detect small to medium effects; (very) small effects might not have been detected.

### Procedure

The data was part of a preregistered three-wave longitudinal study (https://osf.io/6ygcu/?view_only=c77cfb46028447bdb7844cd2c76237aa for the pre-registration^2^; SOM.1 for the links between the paper and the pre-registration as well as deviations from the pre-registration): Time 1 in March 2020 (1 week before school closure; *N* = 112), time 2 in June 2020 (during school closure; *N* = 73), and time 3 in February 2021 (~1 year after school closure; *N* = 73). Time 1 data was intended to serve as a baseline measure for another project that had to be postponed due to the COVID-19 pandemic; this data is thus only used for this project. The data at time 3 will also serve as (part of) the baseline data of the postponed project. After obtaining ethical clearance from all respective parties, adolescents filled out a paper-and-pencil questionnaire during class hours at time 1 and 3, and an online questionnaire at time 2 (via the schools’ online learning platform). At time 1, the COVID-19 virus was starting to spread, but almost no containment measures were in place yet. It was still unclear that schools would close at the time of the data collection. Data was collected on Wednesday through Friday. In the following weekend, the government unexpectedly decided that schools would remain closed from Monday onwards. At time 2, pupils were incentivized through a raffle ticket to boost participation. They were either completely at home (first-year classes; *N* = 90) or attending school once a week (second-year classes; *N* = 34). The latter group had only gone to school three times since the start of the school closure. It was determined by the school which classes were allowed to go back to school. At the start of the school closure, pupils were mainly doing repetition exercises; some pupils had difficulty taking part in school activities as they did not have a computer available at home yet. Around the time of data collection, schools ensured that all pupils had access to a computer. Teachers pre-recorded lectures that pupils were expected to watch at their own pace. Moreover, pupils did exercises by themselves, and answer keys were provided via the online learning platform. Teachers also created quizzes and offered live online Q&A sessions. Pupils therefore mainly worked on their own, with limited access to classmates or teachers; this was mainly done because some families did not have sufficient available computers to allow pupils to be online at a particular time. It should be noted that pupils did not receive online or distance education before school closure. At time 3, only the first-year classes could be followed up. Second year pupils (Year 3 at time 3) transitioned from lower to middle secondary school in the Belgian educational system, accompanied by changes in teaching staff. Therefore, it was not possible to follow up on this group. First-year classes were back to school full-time at the time of the data collection, since schools had fully reopened again. However, several other containment measures were still in place.

### Measures

#### School belonging

It was assessed with four items (T1, T2, T3; e.g., ‘I am proud to be a pupil of this school’; Goodenow, 1993; Phalet et al. 2018; Wang et al. 2011; α_T1_ = 0.81; α_T2_ = 0.75; α_T3_ = 0.83) on a scale from 1 (*totally untrue*) to 5 (*totally true*).

#### School engagement

It was assessed on a scale from 1 (*totally untrue*) to 5 (*totally true*) with 9 items tapping into emotional engagement (e.g., ‘I feel good in class’), behavioral engagement (T1, T2, T3; e.g., ‘I work as hard as I can in class’), and behavioral disaffection (e.g., ‘In class I am easily distracted; Phalet et al. 2018; Skinner et al. 2008; α_T1_ = 0.75; α_T2_ = 0.78; α_T3_ = 0.74). Exploratory factor analyses yielded only one factor, so the composite score consisted of all 9 items.

#### Academic self-esteem

It was assessed with four items (T1, T2, T3; e.g., ‘I feel as smart as others in my class’; Heatherton and Polivy, 1991; Phalet et al. 2018; α_T1_ = 0.62; α_T2_ = 0.69; α_T3_ = 0.71) on a scale from 1 (*totally untrue*) to 5 (*totally true*). Since internal consistencies at T1 and T2 were below conventional thresholds (α < 0.70), confirmatory factor analyses (CFAs) were conducted. The CFAs showed one underlying factor with strong factor loadings (between 0.64 and 0.78 at T1 and between 0.63 and 0.77 at T2).

#### Quality of online instruction

The scale (T2) was newly developed for this study. It originally consisted of 9 items and multiple exploratory factor analyses were run to converge on a composite scale (see SOM.2 for more details). The final scale consisted of four items (e.g., ‘How often did your teachers review or correct your exercises or where they corrected collectively?’) that loaded strongly onto one single factor (factor loadings between 0.61 and 0.72; α = 0.60). As two items were assessed on a scale from 1 (*Never*) to 5 (*Every day*) and two items on a scale from 1 (*Never*) to 4 (*Always*), the items were rescaled (from 0 to 1) before creating a composite score.

#### Teacher support

It was assessed using 4 items (T2; e.g., ‘How often do your teachers encourage you?’) on a scale from 1 (*Never*) to 4 (*Always*; Brondolo et al. 2005; Murray and Greenberg, 2000; Phalet et al. 2018; α = 0.73).

#### Teacher rejection

It was assessed using 4 items (T2; e.g., ‘How often do your teachers expect you cannot do anything right?’) on a scale from 1 (*Never*) to 4 (*Always*; Brondolo et al. 2005; Murray and Greenberg, 2000; Phalet et al. 2018; α = 0.56). Since the internal consistency was below conventional thresholds, a CFA was conducted. This showed one underlying factor with strong factor loadings (between 0.53 and 0.81).

#### Family support with homework

It was assessed with 6 items (T2; e.g., ‘My parents, sibling(s), or someone else at home encouraged me to work hard at school’; Kalter et al. 2014; Phalet et al. 2018) on a scale from 1 (*totally untrue*) to 5 (*totally true*; α = 0.83).

#### Resources at home

The scale consisted of 6 yes/no items (T2; i.e., whether pupils had their own space to study, had their own computer (laptop, tablet) and internet to do their homework, had a garden or terrace, had enough space, had enough time to do homework, and whether they sometimes used their smartphone to study online—the latter item was reverse coded). The number of times adolescents said ‘yes’ were summed to create an overall score.

#### Ethnic origin

A dummy distinguished between ethnic majority and ethnic minority adolescents (as reference) based on self-reported parentage (i.e., at least one (grand)parent born abroad). Ethnic origin was assessed at T1, but information from other times was used when information at T1 was missing.

#### Subjective SES

It was assessed using a SES ladder (T1). Adolescents were told the top of the ladder represented people who were the best off in Belgium, whereas the bottom represented those who were worst off. They then ranked their own family on a scale from 1 (*worst off*) to 10 (*best off*; Adler et al. 2000).

#### Control variables

Analyses were controlled for age, gender, and school track in all models. Only the significant control variables were kept in the final models. Preregistered differences between adolescents with and without higher educated parents were tested. Since this did not make a difference, they were removed from the final models.

### Analytic Plan

The data analyses consisted of several parts. First, this study examined whether school adjustment (H1) declined from before school closure (T1) to during school closure (T2); and explored changes in school adjustment after a year (T3). It also examined whether adolescents from ethnic minority and lower SES backgrounds showed stronger decreases (H3.1). Repeated measures ANOVAs as the main analyses were preregistered with Latent Growth Curve Modelling (LGCM) as a robustness check. However, it was not possible to conduct them due to the high amount of missings and the non-linear growth. The analytic plan was therefore revised and mean comparisons were conducted. Next, lagged regression analyses tested for differences across adolescents’ ethnic and SES backgrounds. Lagged regression analyses control for initial differences in school adjustment. They therefore allow to examine predictors of the difference or change in school adjustment during (T2 outcome – T1 outcome) and after (T3 outcome - T2 outcome) school closure (Adachi and Willoughby, 2015). This analysis was not preregistered.

Second, preregistered lagged regression analyses were conducted to examine the role of school and home contexts for changes in adolescents’ school adjustment outcomes (H2); and whether effects varied according to ethnic origin and SES (H3.2). A main effects model was run with five variables concerning school and home contexts (i.e., quality of online instruction, teacher support, teacher rejection, resources at home, and family support with homework) as well as ethnic origin and subjective SES as predictors. School adjustment at T2 was predicted while taking initial differences into account (i.e., controlling for T1 outcomes). This was followed by a moderation model including interactions between the five contextual predictors on the one hand, and ethnic origin and subjective SES on the other hand. Only significant interactions were retained and significant covariances between predictors, moderators, and outcomes at T1 were added. School adjustment was defined at the latent level, indicated by school belonging, school engagement, and academic self-esteem.

Additionally, mediation analyses were conducted (H3.3.)—which were not preregistered. Ethnic origin and subjective SES were used as predictors, aspects of school and home contexts as mediators, and school adjustment at T2 as an outcome (i.e., controlled for school adjustment at T1). To explore the long-term consequences of school closure, this mediation model was then extended by examining the stability path from T2 to T3 (during school closure to after re-opening)^3^. Finally, it was preregistered to analyze the role of school and home contexts for changes in adolescents’ psychological adjustment as exploratory analyses. The results of these exploratory analyses can be found in SOM.3. All analyses were conducted using M*plus* version 8.6 (Muthén and Muthén, 1998–2017). The sample size might be too small to detect (very) small effects (see under participants). In line with previous studies (Shi et al. 2022), 0.05 < *p* < 0.10 is therefore cautiously interpreted for the hypothesized effects. Missing data were handled using Full Information Maximum Likelihood (FIML) estimation; FIML uses available data without imputing missing data and is therefore an efficient and unbiased method (Enders and Bandalos 2009).

## Results

Descriptive statistics and correlations can be found in Table 1. Measurement invariance across time was assessed for all school adjustment outcomes separately. Scalar invariance (i.e., both equal factor loadings and item intercepts across groups) was established for all constructs (see SOM.4). This implies that all scales showed a similar meaning of items across different timepoints.

**Table 1:** Descriptive statistics and correlations

	1	2	3	4	5	6	7	8	9	10	11	12	13	14	15	16
1																
2	0.57***															
3	0.60***	0.73***														
4	0.32**	0.17	0.10													
5	0.20	0.26*	0.31*	0.61***												
6	0.42***	0.27	0.37**	0.62***	0.71***											
7	0.43***	0.37**	0.22	0.57***	0.32*	0.49***										
8	0.27*	0.36**	0.27	0.47***	0.50***	0.38*	0.48***									
9	0.27*	0.30*	0.31**	0.39**	0.21	0.46***	0.63***	0.40**								
10	−0.04	0.21	0.10	0.11	0.22	0.13	−0.02	−0.10	0.01							
11	0.20	0.20	−0.07	0.34**	0.34**	0.42**	0.28*	0.26*	0.37*	0.17						
12	0.13	0.17	0.08	0.18	0.07	−0.04	0.20	0.27*	0.08	−0.10	0.26*					
13	0.31*	0.43***	0.33*	0.35**	0.23	0.21	0.44***	0.32**	0.33*	0.35**	0.44***	0.24				
14	−0.19	−0.37**	−0.09	−0.22	−0.25*	−0.25	−0.40**	−0.43***	−0.38*	0.19	−0.38**	−0.35**	−0.24			
15	0.19*	0.38**	0.18	0.21*	0.29*	0.26*	0.08	0.33**	0.09	−0.30*	0.37**	0.25	0.17	−0.40**		
16	0.33**	0.29*	0.01	0.26*	0.24	0.19	0.16	0.29	0.10	−0.01	0.10	0.31	0.20	−0.04	0.01	
M	3.75	3.83	3.67	3.43	3.40	3.32	3.39	3.49	3.43	0.40	4.00	4.73	2.75	1.31	52.1	7.84
SD/%	0.75	0.70	0.79	0.53	0.57	0.58	0.67	0.72	0.74	0.19	0.65	0.97	0.62	0.40		1.73
*N* missing	1	0	0	1	2	0	0	3	0	8	8	10	5	6	7	36

1. School belonging T1, 2. School belonging T2, 3. School belonging T3, 4. School engagement T1, 5. School engagement T2, 6. School engagement T3, 7. Academic self-esteem T1, 8. Academic self-esteem T2, 9. Academic self-esteem T3, 10. Quality of online instruction, 11.Family support with homework, 12.Resources at home, 13.Teacher support, 14.Teacher rejection, 15.Ethnic majority (vs. ethnic minority), 16.Subjective SES

*p < 0.05, **p < 0.01, ***p < 0.001

### Attrition Analyses

First, adolescents who participated in all three waves (35.5%) were compared to those who missed at least one wave of data collection (i.e., 18.5% participated only in wave one, 5.6% participated only in wave two, 3.2% participated only in wave three, 16.9% participated only in wave one and two, 19.4% participated only in wave one and three, 0.8% participated only in wave two and three). Adolescents who missed at least one wave of data collection were older on average (*F* (1, 115.82) = 16.35, *p* < 0.001), more often from ethnic minority backgrounds (*χ*^2^ (1) = 10.85, *p* = 0.001), and in vocational tracks (*χ*^2^ (1) = 21.19, *p* < 0.001). They did not differ on subjective SES, parental education, gender, or school adjustment at T1. Second, as most of the models use only T1 and T2 data, selective attrition for Time 1 and 2 was also assessed. Adolescents who participated in both waves (54.2%) were compared to those who participated in only one wave. This indicated that ethnic minority adolescents were more likely to miss one wave of data collection (*χ*^2^ (1) = 11.24, *p* < 0.001). There were no differences based on age, school track, subjective SES, parental education, gender, or school adjustment at T1.

### Changes in School Adjustment

Estimated mean comparisons were first conducted between all school adjustment indicators separately at T1 and T2, T2 and T3, and T1 and T3. Contrary to H1, none of the mean comparisons were significant (all *p*s > 0.10). Differences in average school belonging, school engagement, and academic self-esteem over time could therefore not be established. As a next step, lagged regression analyses were conducted predicting changes in school adjustment during school closure (T2 controlling for initial differences in the outcome at T1) from ethnic origin and subjective SES (see SOM.5 for the full models). In line with H3.1., all school adjustment outcomes were differently predicted by ethnic origin (ethnic majority vs. ethnic minority youth as the reference; for school belonging: *B* = 0.40, SE = 0.16, *β* = 0.28, *p* = 0.012; for school engagement: *B* = 0.32, SE = 0.13, *β* = 0.27, *p* = 0.011; for academic self-esteem: *B* = 0.41, SE = 0.16, *β* = 0.29, *p* = 0.013). For *school belonging*, ethnic minority adolescents started at an estimated average of 3.59 (T1), developed to 3.45 (T2), and ended up at 3.50 (T3). Ethnic majority adolescents started at an estimated average of 3.87 (T1), developed to 4.01 (T2), and ended up at 3.78 (T3). Estimated mean comparisons between both groups showed that at T1 - 1 week prior to school closure—ethnic minority adolescents already showed lower school belonging (Wald *χ*^2^ (1) = 4.07, *p* = 0.044, Cohen’s D = 0.42). These differences were augmented (Wald *χ*^2^ (1) = 9.96, *p* = 0.002, Cohen’s D = 0.63) during school closure at T2. At T3 – 1 year later when schools reopened—the means did not differ anymore (*p* > 0.10).

For *school engagement*, ethnic minority adolescents showed an estimated average of 3.31 (T1), moved to 3.17 (T2), and ended up at 3.13 (T3), whereas ethnic majority adolescents showed an estimated average of 3.53 (T1), moved to 3.52 (T2), and ended up at 3.42 (T3). Estimated mean comparisons showed that ethnic minority adolescents already showed lower school engagement at T1 - 1 week prior to school closure (Wald *χ*^2^ (1) = 4.86, *p* = 0.028, Cohen’s D = 0.41). These differences became larger during school closure at T2 (Wald *χ*^2^ (1) = 6.08, *p* = 0.014, Cohen’s D = 0.66), and were reduced again at T3 – 1 year later when schools reopened (Wald *χ*^2^ (1) = 4.24, *p* = 0.040, Cohen’s D = 0.46).

For *academic self-esteem*, ethnic minority adolescents had an estimated average score of 3.33 (T1), moved to 3.20 (T2), and ended up at 3.37 (T3). Ethnic majority adolescents had an estimated average score of 3.43 (T1), moved to 3.69 (T2), and ended up at 3.48 (T3). Estimated mean comparisons showed that the means of academic self-esteem did not differ significantly at T1 - 1 week prior to school closure (*p* > 0.10). However, differences appeared during school closure at T2 (Wald *χ*^2^ (1) = 7.77, *p* = 0.005, Cohen’s D = 0.71); and they disappeared again when schools reopened at T3 (*p* > 0.10). In line with H3.1., differences in school adjustment between ethnic minority and ethnic majority adolescents were therefore augmented or appeared during school closure, whereas they reduced or disappeared again 1 year later when schools reopened. Importantly, several other containment measures were still in place at T3. Changes in school adjustment thus followed the same pattern as school closures despite other containment measures.

It was also explored whether ethnic origin and subjective SES were significant predictors of school adjustment after re-opening the schools (T3; controlling for school adjustment during school closure at T2). Even though it did not reach significance, the effect of subjective SES was in the expected direction for school belonging (*B* = −0.12, SE = 0.07, *β* = −0.26, *p* = 0.078): lower subjective SES was associated with increased school belonging after reopening the schools (vs. during school closure).

### The Role of School and Home Contexts, Ethnic Origin, and SES

#### Main effects

First a lagged regression model was run. School adjustment comprised of a latent factor with school belonging (standardized factor loading at T1: 0.50; standardized factor loading at T2: 0.61), school engagement (standardized factor loading at T1: 0.65; standardized factor loading at T2: 0.44), and academic self-esteem (standardized factor loading at T1: 0.89; standardized factor loading at T2: 0.53) as indicators. Importantly, initial differences were taken into account by controlling for school adjustment 1 week before school closure (T1); this allows to predict *changes* in school adjustment during school closure (T2). School adjustment at T2 was regressed on the factors in the school and home context at T2, ethnic origin, and subjective SES (CFI = 0.91, RMSEA = 0.06). Results can be found in Table 2. In line with H2, higher quality online instruction (e.g., correcting exercises together) and less experienced rejection from teachers protected school adjustment during school closure across all adolescents (i.e., while taking initial differences into account). Additionally, youth with an ethnic minority background reported decreased school adjustment during school closure compared to their peers with an ethnic majority background. This confirms the analysis with school adjustment as separate outcomes above.

**Table 2:** Moderation models predicting school adjustment during school closure

	Main effects *B* (SE) *β*	Interaction *B* (SE) *β*
School adjustment at T1	0.32 (0.14)^*^ 0.44	0.32 (0.13)^*^ 0.45
Majority (vs. minority)	0.26 (0.09)^**^ 0.52	0.22 (0.08)^**^ 0.47
Subjective SES	0.02 (0.02) 0.16	0.02 (0.02) 0.12
Quality of online instruction	0.60 (0.23)^**^ 0.46	0.45 (0.21)^*^ 0.37
Family support with homework	−0.09 (0.05) −0.22	−0.09 (0.05)^+^ −0.24
Resources	−0.02 (0.03) −0.07	−0.02 (0.03) −0.07
Teacher support	0.07 (0.06) 0.18	0.19 (0.08)^*^ 0.46
Teacher rejection	−0.29 (0.11)^**^ −0.44	−0.27 (0.10)^**^ −0.44
Teacher support X majority	–	−0.19 (0.09)^*^ −0.37

The main effects and interaction models were ran separately with school adjustment at T2 as DV. School adjustment was a latent variable with school belonging, school engagement, and academic self-esteem as indicators. Effects were standardized using STDYX standardization

^+^*p* < 0.10; ^*^p < 0.05; ^**^p < 0.01

#### Moderation

**Fig. 1:**
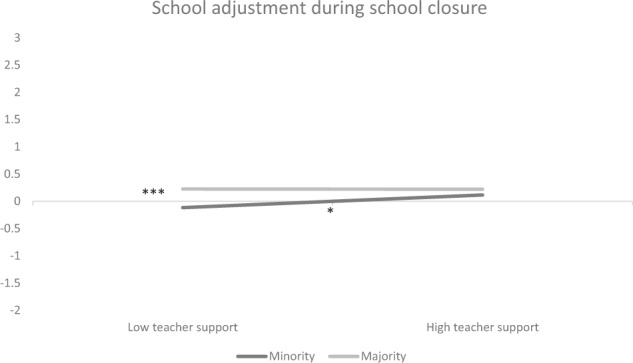
*Interaction between teacher support and ethnic origin predicting school adjustment during school closure*. ^+^*p* < 0.10, ^*^p < 0.05, ^**^p < 0.01, ^***^p < 0.001

It was then tested whether the effects of the factors in the school and home context depended on adolescents’ ethnic origin and subjective SES (CFI = 0.90, RMSEA = 0.06; Table 2). A significant interaction between teacher support and ethnic origin was found (Fig. 1). Experienced teacher support only made a difference for adolescents with an ethnic minority background. Those who experienced lower teacher support (−1SD) reported significantly lower school adjustment during school closure compared to those experiencing higher teacher support (+1 SD; Wald χ^2^ (1) = 5.62, *p* = 0.018). Moreover, at lower levels of teacher support, they showed significantly lower school adjustment during school closure compared to their ethnic majority peers (Wald *χ*^2^ (1) = 11.54, *p* < 0.001); the difference was not significant at higher levels of teacher support (+1 SD; *p* > 0.10). Importantly, these effects emerged while taking initial differences 1 week before school closure into account. Thus, in line with H3.2., teacher support protected school adjustment during school closure among ethnic minority youth, so that adjustment gaps were not augmented at high levels of teacher support.

#### Mediation

**Fig. 2:**
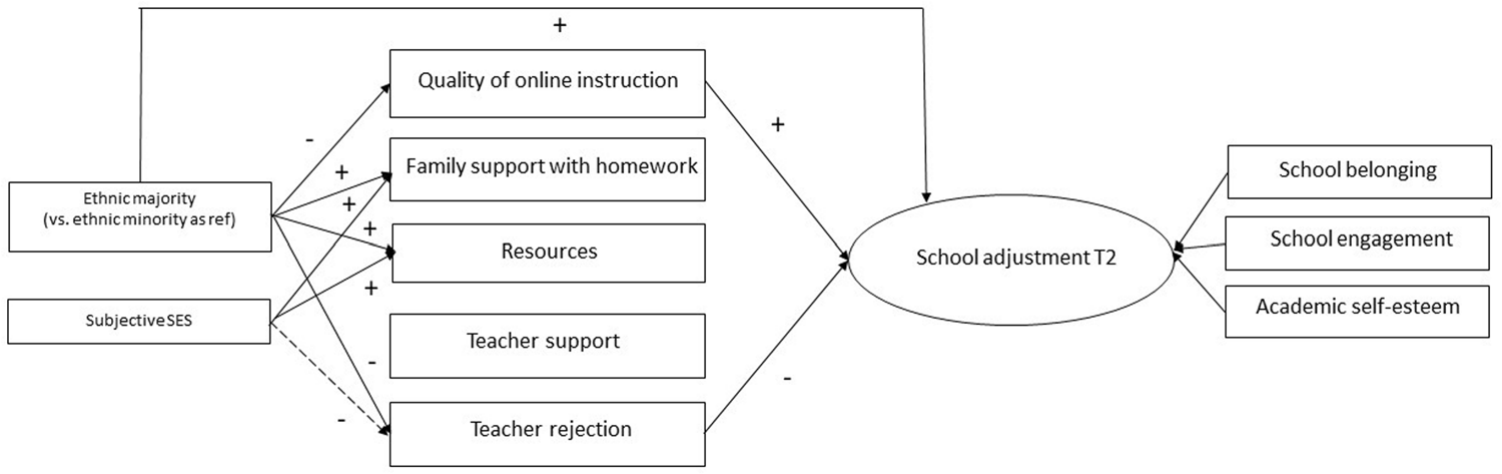
*Mediation model predicting school adjustment during school closure*. Only significant paths are displayed. Dashed lines indicate 0.05 < *p* < 0.10. School adjustment was a latent variable with school belonging, school engagement, and academic self-esteem as indicators

Not-preregistered mediation analyses were additionally conducted to examine whether differences in school adjustment could be explained by differences in school and home contexts (CFI = 0.96, RMSEA = 0.05; Table 3 and Fig. 2). Results confirmed restrictions in school and home contexts of adolescents from ethnic minority and lower SES backgrounds. In line with H3.3., both reported less family support with homework, fewer resources at home, and more teacher rejection (0.05 < *p* < 0.10 for subjective SES). However, contrary to the hypothesis, ethnic minority youth also reported *higher* quality online instruction during school closure compared to their ethnic majority peers. In turn, only higher quality of online instruction and less teacher rejection protected school adjustment during school closure (i.e., while taking initial differences 1 week before school closure into account). Thus, in line with H3.3., ethnic minority youth showed worse school adjustment during school closure via more teacher rejection (*B* = 0.16, SE = 0.07, *β* = 0.18, *p* = 0.017 for the indirect effect) compared to their ethnic majority peers. Arguably, adolescents from lower SES backgrounds would also show worse school adjustment via more teacher rejection, but the indirect effect remained non-significant. This is probably due to the weaker effect of subjective SES on teacher rejection. Contrary to the expectation, adolescents from ethnic minority backgrounds also showed better school adjustment during school closure via experiencing higher quality online instruction (*B* = −0.15, *SE* = 0.07, *β* = −0.16, *p* = 0.038 for the indirect effect). Importantly, both indirect effects cancelled each other out, so that the total indirect effect from ethnic origin to school adjustment at T2 was not significant (*B* = −0.06, SE = 0.11, *β* = −0.06, *p* = 0.596). Thus, higher quality online instruction compensated for the higher teacher rejection ethnic minority youth experienced, although the total effect of ethnicity remained significant (*B* = 0.35, SE = 0.14, *β* = 0.39, *p* = 0.011).

**Table 3:** Mediation model predicting school adjustment during school closure

	Quality of online instruction *B (SE)* β	Family support with homework *B (SE)* β	Resources *B (SE)* β	Teacher support *B (SE)* β	Teacher rejection *B (SE)* β	School adjustment *B (SE)* β
School adjustment at T1	–	–	–	–	–	0.48 (0.28)^+^ 0.51
Majority (vs. minority)	−0.13 (0.05)^**^ −0.34	0.45 (0.16)^**^ 0.34	0.51 (0.24)^*^ 0.26	0.16 (0.15) 0.13	−0.32 (0.09)^**^ −0.40	0.41 (0.14)^**^ 0.45
Subjective SES	−0.00 (0.02) −0.03	0.10 (0.05)^*^ 0.28	0.18 (0.08)^*^ 0.33	0.07 (0.05) 0.21	−0.05 (0.03)^+^ −0.23	0.03 (0.05) 0.11
Quality of online instruction	–	–	–	–	–	1.12 (0.36)^**^ 0.47
Family support with homework	–	–	–	–	–	−0.16 (0.10) −0.23
Resources	–	–	–	–	–	−0.03 (0.06) −0.07
Teacher support	–	–	–	–	–	0.10 (0.15) 0.13
Teacher rejection	–	–	–	–	–	−0.50 (0.15)^**^ −0.45

The mediation model was ran in one model, whereby quality of online instruction, family support with homework, resources, teacher support, and teacher rejection served as mediators, and school adjustment at T2 as DV. School adjustment was a latent variable with school belonging, school engagement, and academic self-esteem as indicators. Effects were standardized using STDYX standardization

^+^*p* < 0.10, ^*^p < 0.05, ^**^p < 0.01

As a next step, potential long-term implications were explored by running the same mediation model, but this time including a path from T2 school adjustment to T3 school adjustment (not preregistered; CFI = 0.94, RMSEA = 0.05). The direct effects that significantly improved the model fit were retained (from school adjustment at T1 to T3, *B* = 0.44, SE = 0.21, *β* = 0.52, *p* = 0.041). In terms of long-term implications of school closures, a significant positive stability path from school adjustment during the school closure (T2) to 1 year beyond was found (T3; *B* = 0.49, SE = 0.20, *β* = 0.55, *p* = 0.013). This was the case over and beyond initial differences in school adjustment before school closure (i.e., while controlling for T1). Moreover, the significant indirect paths from ethnic origin to school adjustment at T2 via teacher rejection cautiously extended until a year after school closure when schools had reopened again (*B* = 0.08, SE = 0.04, *β* = 0.10, *p* = 0.058). For the quality of online instruction, it did not extend until a year after (*p* > 0.10). In other words, ethnic minority adolescents experienced more teacher rejection during school closure compared to their ethnic majority peers. This was in turn associated with lower school adjustment not only during school closure, but also 1 year later. Thus, the impact of at least one aspect of the school context, i.e., student-teacher relationship quality, was not limited to the period of school closure, but also extended until 1 year beyond after schools had reopened again. Moreover, the effect of ethnic origin on school adjustment at T2 also showed indications to extend until a year after school closure (from ethnic origin to T3 school adjustment via T2 school adjustment; *B* = 0.18, SE = 0.10, *β* = 0.22, *p* = 0.078). Ethnic minority adolescents thus reported lower school adjustment compared to their majority peers during school closure and 1 year later. So other factors not captured in this study might have explained their disadvantage.

## Discussion

The present study contributes to an emerging line of research examining the consequences of the COVID-19 pandemic on adolescents’ adjustment (Branje and Sheffield Morris, 2021). It also goes beyond previous work by examining whether adolescents’ school adjustment worsens during school closures compared to 1 week before and recovers after 1 year; and whether school and home contexts can act as a contextual buffer against potentially negative consequences of school closures. Moreover, it adds to the current knowledge by investigating whether these changes in adjustment and contextual buffers vary across adolescents from different ethnic and SES backgrounds. This longitudinal study flashed out the negative consequences of school closures for adolescents’ school adjustment. It also showed the important role of teachers to buffer against this, particularly for adolescents from ethnic minority and lower SES backgrounds.

### Changes in School Adjustment Following School Closure

Contradictory to H1, this study could not establish general decreases in school adjustment during school closure. Arguably, there is significant variation in how adolescents cope with school closures. Previous research has found that some adolescents actually seem to fare well by school closures whereas others are much harder affected (Salmela-Aro et al. 2021). For example, some adolescents might engage more easily with their schoolwork at home than others depending on their social environments. Overall, these different individual pathways may balance each other out. This might explain why the results could not establish an average change in school adjustment during and following school closure across all adolescents. Alternatively, changes in school adjustment could have been small in such a short period of time; and the sample size of the present study might have been too small to detect them.

Instead of general changes in school adjustment, this study showed that the consequences of school closures depended on adolescents’ ethnic and SES backgrounds. In support of H3.1., school closure augmented already existing school adjustment gaps between ethnic minority adolescents and their ethnic majority peers (Heath and Brinbaum, 2014). Previous research already indicated reduced school adjustment particularly among adolescents from ethnic minority (e.g., Paizan et al. 2021) and lower SES backgrounds (e.g., Easterbrook et al. 2022). The current findings corroborate and extend these earlier findings. They demonstrate that gaps in school belonging, school engagement, and academic self-esteem between ethnic minority and ethnic majority youth were augmented or appeared during school closure. This was the case despite the brief time interval between the two time points at pre- and during school closure (i.e., only 3 months). The findings did not show significant SES-based differences during school closure. Still, there were also indications that reopening the schools benefitted the school adjustment of youth with a lower subjective SES more. Moreover, the results demonstrate that ethnicity-based gaps in school adjustment reduced or disappeared again after reopening the schools.

Embedding these findings within the COVID-19 containment measures at the time in Belgium, there were almost no containment measures in place 1 week before school closure (T1); schools closed one day to the next. At T2, a full lockdown was in place including the closure of schools. Schools were fully open again 1 year after school closure (T3). No school closures happened between the second and third wave of data collection (except for a prolonged Fall break). Still, other containment measures were in place at T3, including the closure of non-essential shops, bars, and restaurants. Although these measures were not lifted yet, the results showed that school adjustment gaps reduced or disappeared at T3. It is thus unclear whether increasing gaps at T2 were solely due to school closures or due to a combination of COVID-19-related factors including other containment measures. Still, for the reduction in gaps at T3, it is most likely that schools reopening was the driving factor. School belonging, school engagement, and academic self-esteem are precursors of later school achievement (Gillen-O’Neel and Fuligni, 2013). The findings of the present study might therefore (partially) explain increased achievement gaps during the COVID-19 pandemic. School closures might therefore have important long-lasting consequences via reduced achievement; and they may maintain or augment already existing inequalities.

### The Role of School and Home Contexts

The present findings not only demonstrated the consequences of school closures but also highlighted protective mechanisms in the school context. In line with H2, adolescents who received higher quality of online instruction and experienced less teacher rejection reported better school adjustment during school closure; and this was the case while taking initial differences in school adjustment prior to school closure into account. In line with a dynamic systems theory of development (Thelen and Smith, 2006), different social interactions may lead to different developmental pathways. Research on the protective and risk factors in development posits that positive relationships with peers (Grew et al. 2022) or teachers (McGrath and Van Bergen, 2015) can protect adolescents against various stressors. Accordingly, positive teacher-pupil interactions during school closures seem to sustain adolescents’ school adjustment. Both components reflect different aspects of teacher-pupil interactions. Quality of online instruction refers to more instrumental support during school closure. Quality of teacher-pupil relationships refers to a more affective component of teacher-pupil interactions. Previous research already established the important role of teacher rejection (and support) for adolescents’ school adjustment (Roorda et al. 2011). The current findings extend these findings by looking at *changes* in school adjustment from before to during school closures during the COVID-19 pandemic; and they demonstrate the important complementary and additive effect of instrumental and affective components of teacher-pupil interactions during school closures.

While teacher-student interactions are key to the school adjustment of all adolescents, they are particularly important for adolescents from ethnic minority backgrounds. In line with H3.2, high teacher support protected school adjustment during school closures especially among ethnic minority youth. However, none of the other interactions were significant. This implies that processes work rather similar across adolescents from different ethnic and SES backgrounds. The quality of the teacher-pupil relationship might be particularly prone to show different effects among ethnic minority and ethnic majority youth. Teachers generally have an ethnic majority background. Consequently, teacher-pupil relationships have a distinct intergroup component for ethnic minority youth (Baysu et al. 2021). Feeling support from the teacher might signal identity valuation and inclusion in school to ethnic minority youth; and it might therefore buffer against negative school adjustment consequences during school closures (Baysu et al. 2021). The quality of online instruction might on the contrary be less susceptible to perceived differential treatment by teachers, because it relates to perceptions of instrumental support (at a group-level) such as correcting exercises together or what you learn at school. Similarly, these processes might be less pronounced among youth from lower SES backgrounds, whose disadvantaged backgrounds are relatively less visible (but see SOM.3 for significant interactions between teacher rejection and SES on psychological adjustment).

Interestingly, this study did not find any effects of the home context (i.e., family support with homework and resources at home) when taking the school context into account (but see SOM.3 for significant interactions between family support with homework and SES on psychological adjustment). Whereas previous studies found that the home context plays a critical role during school closures (e.g., Paizan et al. 2021), they did not simultaneously take the school context into account. It seems that schools, and teachers in particular, play a more important role in sustaining adolescents’ school adjustment during school closures. This is in line with the idea that contextual factors often have domain-specific effects (Benner and Graham, 2013). Accordingly, school-related factors seem to predict school adjustment more strongly than home-related factors. Teachers might serve as a critical bridge between school and pupils during school closures; and they might therefore secure adolescents’ school adjustment regardless of the home context. Future research should incorporate factors in both school and home contexts to shed further light on which factors can buffer against the adverse consequences of school closures.

Although school and home contexts related relatively similar to adolescents’ school adjustment, adolescents from ethnic minority and lower SES backgrounds were less likely to reside in supportive environments during school closures. In line with H3.3., adolescents from ethnic minority and lower SES backgrounds were less likely to receive support from family and teachers and had fewer resources. These contexts had in turn negative consequences for their school adjustment during school closures. Importantly, adolescents were all in the same school with the same teachers. These differences can therefore not be attributed to school-level variation, but rather point toward perceptions of differential treatment based on ethnic and SES origin. Although positive contexts benefit adolescents from different ethnic and SES backgrounds similarly, less optimal contexts pose them at risk of more adverse developmental outcomes during school closures.

However, contradictory to the hypothesis, ethnic minority adolescents also reported *higher* quality of online instruction than their ethnic majority peers; and this protected their school adjustment during school closure. Teachers may be aware of pupils’ lack of resources or difficult situation at home (Kim and Asbury, 2020). Consequently, they may try to provide instrumental support as reflected by higher quality of online instruction. This is also supported by the fact that quality of online instruction was higher among adolescents in vocational (vs. academic) school tracks, where ethnic minority youth are overrepresented (Baysu et al. 2018). Classes in vocational tracks are generally smaller. It might therefore have been easier for teachers to provide high-quality online instruction to vulnerable pupils in these classes (vs. larger classes in academic tracks). These findings combined demonstrate that adolescents from disadvantaged backgrounds might be more likely to benefit from positive school (and home) contexts but might also be lacking some critical support structures (Baysu et al. 2021). Although ethnic minority youth received higher quality of online instruction, this only compensated their higher experienced rejection from teachers. They still showed poorer school adjustment compared to their ethnic majority peers during school closure nevertheless, even when taking into account initial differences in their school adjustment before school closure. Moreover, these effects also endured until (at least) 1 year later, so reopening the schools did not fully recover some of the damage that had been done.

Overall, results were more pronounced among youth from ethnic minority backgrounds than from lower SES backgrounds. Measuring SES among adolescents remains a challenge (Svedberg et al. 2016). The present study used two different indicators of adolescents’ SES backgrounds, namely parental education and subjective SES. Although parental education is most commonly used, adolescents are generally not well-equipped to answer these questions (Hammond et al. 2021). The lack of significant findings of parental education in the present study might be attributed to this. Although there were some effects of subjective SES, they were also less pronounced than those of ethnic origin. Interestingly, parental education and subjective SES are not strongly related until late adolescence or young adulthood (Hammond et al. 2021). This points toward developmental changes in understandings of SES. Although more age-appropriate, subjective SES might relate to a separate subdimension in adolescence than more objective indicators like parental education (Svedberg et al. 2016). For example, objective and subjective SES measures were found to relate to adolescents’ substance use in different ways (Hammond et al. 2021). Arguably, early adolescents, like in the present study, might be better equipped to answer questions regarding their ethnic origin than their SES backgrounds. It could be that these effects were consequently more pronounced. Future research should aim to develop new methods to capture adolescents’ SES backgrounds to shed more light on developmental pathways across different SES groups.

### Limitations

This study is not without limitations. First, the sample size was rather small and adolescents came from only one school. The design of the study prevented the addition of participants (and other schools). Post-hoc sensitivity analyses showed that it was possible to detect small to medium effects. Accordingly, it is possible that (very) small effects might not have been detected and were missed out on in the present study. As in all convenience samples, it is unclear how representative or generalizable the findings are. However, the results are very much in line with previous studies that used larger samples and were conducted in different countries. This strengthens the confidence in the current study’s conclusions. Second, the difficult circumstances of the data collection resulted in relatively high attrition rates. This was particularly the case for the three-wave data; and to a lesser extent for the first two waves - that were the focus of most analyses. Attrition analyses showed that ethnic minority youth (and older adolescents and adolescents in vocational school tracks) were more likely to drop out. Since there was no selective attrition in the outcome variables and the findings do not go against the Missing at Random assumption, FIML is an unbiased and robust method to handle these missing data (Enders and Bandalos 2009). Third, some of the measures had a somewhat low internal consistency. The factor structure of all measures was confirmed and showed proper factor loadings. Additionally, most of these measures such as teacher rejection (α between 0.70 and 0.88; Baysu et al. 2021; Brondolo et al. 2005) and academic self-esteem (*α* = 0.92; Heatherton and Polivy, 1991) have been used in previous research and had higher internal consistencies. Their slightly lower internal consistencies in the present study could be due to the smaller sample size (Bujang et al. 2018). Even though the results were meaningful, they should still be interpreted with caution. Finally, the study could not distinguish between different ethnic origin groups. Potentially, adolescents from some origin groups are harder affected by school closures than others (Bayrakdar and Guveli, 2020). This might particularly be the case for those from lower SES backgrounds. Future research should incorporate intersections between adolescents’ ethnic and SES backgrounds to further disentangle their relative and joint contributions.

## Conclusion

Many adolescents were confronted with school closures and had to learn from home. Yet, little is known about how school closures affect their school adjustment and how to protect them from potential adverse consequences. This study used a unique longitudinal design with measurements 1 week before, during, and 1 year after the first school closure in Belgium. It examined how school and home contexts could buffer against the negative impact of school closures among youth from different ethnic and SES backgrounds. The findings show the important negative consequences of school closures for school adjustment in general and for adolescents from ethnic minority (and lower SES) backgrounds in particular. They also show the critical role of teachers in preserving adolescents’ school adjustment during school closures. Teachers are often aware of adolescents who are at risk during school closures and the importance of maintaining contact (Kim and Asbury, 2020). Still, they consider it also more difficult to maintain good relationships during school closures (Chrisman and Alnaim, 2021). This study shows that it is essential to provide both instrumental support and affective teacher-pupil interactions during school closures; and that this is especially important for adolescents from ethnic minority backgrounds. However, adolescents from ethnic minority and lower SES backgrounds also resided in less optimal home and school contexts during school closures, with important implications for their school adjustment. It is thus critical that adolescents stay in touch with their teachers during school closures to protect their school adjustment. In sum, it is critical to advocate social connectedness with teachers, while social distancing from them.

## Supplementary information

Supplementary Information

## Footnotes

1 Year 1 and 2 in secondary school corresponds to middle school in the US.

2 The whole project was preregistered, but not all the research questions were investigated in this paper.

3 As preregistered, it was also examined whether school adjustment at T3 (while controlling for T2) could be predicted from school and home contexts at T2, but this did not show any effects. These models are not reported further, but the results can be obtained from the corresponding author.

## References

[CR1] Achterberg M, Dobbelaar S, Boer OD, Crone EA (2021). Perceived stress as mediator for longitudinal effects of the COVID-19 lockdown on wellbeing of parents and children. Nature Scientific Reports.

[CR2] Adachi P, Willoughby T (2015). Interpreting effect sizes when controlling for stability effects in longitudinal autoregressive models: Implications for psychological science. European Journal of Developmental Psychology.

[CR3] Adler NE, Epel ES, Castellazzo G, Ickovics JR (2000). Relationship of subjective and objective social status with psychological and physiological functioning: Preliminary data in healthy White women. Health Psychology.

[CR4] Ariyo E, Amurtiya M, Yemisi Lydia O, Oludare A, Ololade O, Patience Taiwo A, Abolanle Olukemi L, Ogunniyi D (2022). Socio-demographic determinants of children home learning experiences during COVID 19 school closure. International Journal of Educational Research Open.

[CR5] Bambra C, Lynch J, Smith KE (2021). The unequal pandemic: COVID-19 and health inequalities.

[CR6] Bayrakdar, S., & Guveli, A. (2020). *Inequalities in home learning and schools’ provision of distance teaching during school closure of COVID-19 lockdown in the UK (No. 2020-09)*. Institute for Social and Economic Research Working Paper Series. https://www.iser.essex.ac.uk/research/publications/working-papers/iser/2020-09.

[CR7] Baysu G, Alanya A, de Valk H (2018). School trajectories of the second-generation of Turkish immigrants in Sweden, Belgium, Netherlands, Austria and Germany. International Journal of Comparative Sociology.

[CR8] Baysu G, Hillekens J, Phalet K, Deaux K (2021). How diversity approaches affect ethnic minority and majority adolescents: Teacher-student relationship trajectories and school outcomes. Child Development.

[CR9] Benner AD, Graham S (2013). The antecedents and consequences of racial/ethnic discrimination during adolescence: Does the source of discrimination matter?. Developmental Psychology.

[CR10] Branje S, Sheffield Morris A (2021). The impact of the COVID-19 pandemic on adolescent emotional, social, and academic adjustment. Journal of Research on Adolescence.

[CR11] Brondolo E, Kelly K, Coakley V, Gordon T, Thompson S, Levy E, Contrada RJ (2005). The perceived ethnic discrimination questionnaire. Journal of Applied Social Psychology.

[CR12] Bujang MA, Omar ED, Baharum NA (2018). A review on sample size determination for Cronbach’s alpha test: A simple guide for researchers. Malaysian Journal of Medical Science.

[CR13] Chrisman MS, Alnaim L (2021). Resources needed for education and meal programs by urban school teachers and staff during the 2019 coronavirus pandemic. Journal of School Health.

[CR14] Demirtaş-Zorbaza S, Ergeneb T (2020). School adjustment of first-grade primary school students: Effects of family involvement, externalizing behavior, teacher and peer relations. Children and Youth Services Review.

[CR15] Dziak JJ, Dierker LC, Abar B (2021). The interpretation of statistical power after the data have been gathered. Current Psychology.

[CR16] Easterbrook, M. J., Doyle, L., Grozev, V. H., Kosakowska-Berezecka, N., Harris, P. R., & Phalet, K. (2022) Socioeconomic and gender inequalities in home learning during the COVID-19 pandemic: examining the roles of the home environment, parent supervision, and educational provisions. *Educational and Developmental Psychologist*. Advance online publication. 10.1080/20590776.2021.2014281.

[CR17] Enders CK, Bandalos DL (2009). The relative performance of full information maximum likelihood estimation for missing data in structural equation models. Structural Equation Modeling: A Multidisciplinary Journal.

[CR18] Engels MC, Phalet K, Gremmen MC, Dijkstra JK, Verschueren K (2020). Adolescents’ engagement trajectories in multicultural classrooms: The role of the classroom context. Journal of Applied Developmental Psychology.

[CR19] García Coll C, Crnic K, Lamberty G, Wasik BH, Jenkins R, Vázquez García H, McAdoo HP (1996). An integrative model for the study of developmental competencies in minority children. Child Development.

[CR20] Gillen-O’Neel C, Fuligni A (2013). A longitudinal study on school belonging and academic motivation across high school. Child Development.

[CR21] González S, Bonal X (2021). COVID-19 school closures and cumulative disadvantage: Assessing the learning gap in formal, informal and non-formal education. European Journal of Education.

[CR22] Goodenow C (1993). The psychological sense of school membership among adolescents: Scale development and educational correlates. Psychology in the Schools.

[CR23] Grew E, Baysu G, Turner RN (2022). Experiences of peer victimization and teacher support in secondary school predict university enrolment 5 years later: Role of school engagement. British Journal of Educational Psychology.

[CR24] Hammond MA, Khurana A, Stormshak EA (2021). Adolescent measures of family socioeconomic status: Reliability, validity, and effects on substance use behaviors in adolescence and young adulthood. Preventive Medicine Reports.

[CR25] Heath A, Brinbaum Y (2014). Unequal attainments.

[CR26] Heatherton TF, Polivy J (1991). Development and validation of a scale for measuring state self-esteem. Journal of Personality and Social Psychology.

[CR27] Kalter, F., Heath, A. F., Hewstone, M., Jonsson, J. O., Kalmijn, M., Kogan, I., & Van Tubergen, F. (2014). Children of Immigrants Longitudinal Survey in Four European Countries (CILS4EU)-Full version. Data file for on-site use. GESIS Data Archive, Cologne, ZA5353 Data file Version 1.1.0. 10.4232/cils4eu.5353.1.1.0.

[CR28] Kim LE, Asbury K (2020). ‘Like a rug had been pulled from under you’: The impact of COVID-19 on teachers in England during the first six weeks of the UK lockdown. British Journal of Educational Psychology.

[CR29] Klootwijk CLT, Koele IJ, Van Hoorn J, Güroğlu B, Van Duijvenvoorde ACK (2021). Parental support and positive mood buffer adolescents’ academic motivation during the COVID-19 pandemic. Journal of Research on Adolescence.

[CR30] Koirala, A., Goldfeld, S., Bowen, A.C., Choong, C., Ryan, K., Wood, N., Winkler, N., Danchin, M., Macartney, K., & Russell, F.M. (2021). Lessons learnt during the COVID-19 pandemic: Why Australian schools should be prioritized to stay open. *Journal of Paediatrics and Child Health*. Advance online publication. 10.1111/jpc.15588. 10.1111/jpc.15588 PMC8242752 34101922

[CR31] Lietaert S, Roorda D, Laevers F, Verschueren K, De Fraine B (2015). The gender gap in student engagement: The role of teachers’ autonomy support, structure, and involvement. British Journal of Educational Psychology.

[CR32] Maiya S, Dotterer AM, Whiteman SD (2021). Longitudinal changes in adolescents’ school bonding during the COVID-19 pandemic: Individual, parenting, and family correlates. Journal of Research on Adolescence.

[CR33] Mastrotheodoros, S. (2020). *The Effects of COVID-19 on Young People’s Mental Health and Psychological Well-being*. Youth Partnership: Partnership between the European Commission and the Council of Europe in the field of youth. https://pjp-eu.coe.int/documents/42128013/72351197/Effects-COVID-Youth-Mental-Health-Psychological-Well-Being.pdf.

[CR34] McGrath KF, Van Bergen P (2015). Who, when, why and to what end? Students at risk of negative student–teacher relationships and their outcomes. Educational Research Review.

[CR35] Moguérou L, Santelli E (2015). The educational supports of parents and siblings in immigrant families. Comparative Migration Studies.

[CR36] Murray C, Greenberg MT (2000). Children’s relationships with teachers and bonds with school. Journal of School Psychology.

[CR37] Muthén, L. K., & Muthén, B. O. (1998–2017). *Mplus User’s Guide. Eighth Edition*. Los Angeles, CA: Muthén & Muthén.

[CR38] Paizan, M. A., Benbow, A. E. F., Aumann, L., & Titzmann, P. (2021). Home-learning during COVID-19: The psychological adjustment of minority and majority adolescents. *School Psychology. Advance online publication*. 10.1037/spq0000489. 10.1037/spq0000489 34928642

[CR39] Phalet, K., Meuleman, B., Hillekens, J., & Sekaran, S. (2018). *Leuven-CILS technical report longitudinal 2012–2015*.

[CR40] Romm KF, Won Park Y, Hughes JL, Gentzler AL (2021). Risk and protective factors for changes in adolescent psychosocial adjustment during COVID-19. Journal of Research on Adolescence.

[CR41] Roorda DL, Koomen HMY, Spilt JL, Oort FJ (2011). The influence of affective teacher-student relationships on students’ school engagement and achievement: a meta-analytic approach. Review of Educational Research.

[CR42] Rubin KH, Bukowski W, Parker J, Bowker JC, Damon W, Lerner R (2008). Peer interactions, relationships, and groups. Developmental Psychology: An Advanced Course.

[CR43] Salmela-Aro K, Upadyaya K, Vinni-Laakso J, Hietajärvi L (2021). Adolescents’ longitudinal school engagement and burnout before and during COVID-19 – The role of socio-emotional skills. Journal of Research on Adolescence.

[CR44] Shi K, Yang Y, De Vos J, Zhang X, Witlox F (2022). Income and commute satisfaction: On the mediating roles of transport poverty and health conditions. Travel Behavior and Society.

[CR45] Skinner EA, Kindermann TA, Furrer CJ (2008). A motivational perspective on engagement and disaffection conceptualization and assessment of children’s behavioral and emotional participation in academic activities in the classroom. Educational and Psychological Measurement.

[CR46] Suárez-Orozco C, Motti-Stefanidi F, Marks A, Katsiaficas D (2018). An integrative risk and resilience model for understanding the adaptation of immigrant-origin children and youth. American Psychologist.

[CR47] Svedberg P, Nygren JM, Staland-Nyman C, Nyholm M (2016). The validity of socioeconomic status measures among adolescents based on self-reported information about parents occupations, FAS and perceived SES; implication for health related quality of life studies. BMC Medical Research Methodology.

[CR48] Syed M, Santos C, Yoo HC, Juang LP (2018). Invisibility of racial/ethnic minorities in developmental science: Implications for research and institutional practices. American Psychologist.

[CR49] Thelen E, Smith LB, Damon W, Lerner RM (2006). Dynamic systems theories. Handbook of Child Psychology.

[CR50] Wang MT, Willett JB, Eccles JS (2011). The assessment of school engagement: Examining dimensionality and measurement invariance by gender and race/ethnicity. Journal of School Psychology.

