# Supplementary material for: How School and Home Contexts Impact the School Adjustment of Adolescents from Different Ethnic and SES Backgrounds During COVID-19 School Closures
Source: J Youth Adolesc. 2023 Apr 21;52(8):1549–65. doi: 10.1007/s10964-023-01772-z (PMC10120503; doi:10.1007/s10964-023-01772-z)
Supplement: Supplementary file 1 — Supplementary Information [file 10964_2023_1772_MOESM1_ESM.docx]

**Supplementary Online Materials**

**1. Links to and deviations from the pre-registration**

We preregistered the whole project. Therefore, not all research questions are addressed in the paper. Below we give an overview of which sections in the paper refer to which preregistered research questions.

- Sections about changes in school adjustment refer to RQ1 in the preregistration
- Sections about the role of home and school contexts refer to RQ2 in the preregistration
- Sections about differences based on adolescents' ethnic and SES backgrounds refer to RQ1 and RQ2 in the preregistration

Below we give a similar overview of the study hypotheses.

- H1 refers to H1.1. in the preregistration
- H2 refers to H2.1. in the preregistration
- H3.1. refers to H1.3 in the preregistration
- H3.2. refers to H2.2. in the preregistration
- H3.3. was not preregistered beforehand

Finally, we report the deviations from the pre-registration.

- RQ1 and RQ2 in the preregistration also address psychological adjustment as an outcome. RQ1 examines changes in psychological adjustment; this will be done in another paper (H1.2. and part of H1.3. in the preregistration). RQ2 explores the role of home and school contexts for psychological adjustment (E2.3.a and E2.3.b in the preregistration). These analyses are reported in SOM.3.
- The preregistration mentions that adolescents’ SES backgrounds would be assessed using two indicators: subjective SES and parental education. We tested for preregistered differences between adolescents with and without higher educated parents, but this did not make a difference and was left out of the final models, which is briefly reported and discussed in the paper.
- We planned to conduct repeated measures ANCOVAs with Latent Growth Curve Modelling as a robustness check to test changes in school adjustment. This was not possible due to the high amount of missings and the non-linear growth. We instead conducted mean comparisons, followed by lagged regression analyses to test for differences across adolescents’ ethnic and SES backgrounds.
- For the long-term effects of school closures, we examined whether we could predict school adjustment at T3 (while controlling for T2) from school and home contexts at T2 as preregistered, but we did not find any effects. We additionally extended the mediation model by a stability path from T2 to T3 which was not preregistered.
- The mediation hypothesis was not preregistered, therefore also the mediation model was not preregistered. We added this hypothesis and model since we (in hindsight) argued that ethnicity- and SES-based differences could play out in two different ways, as explained in the paper.

**2. Development of a new scale for the quality of online instruction**

As preregistered, we started with 9 items in total. Items were as follows:

- ‘What percentage (%) of your teachers gave online tasks, exercises, or lectures to make you learn?’ on a scale from 0% (= Nobody) to 100% (= All teachers)
- ‘What percentage (%) of the subject matter you received was new?’ on a scale from 0% (= Everything was repetition) to 100% (= Everything was new)
- ‘How often did you receive online tasks, exercises or lectures?’ on a scale from 1 (= Never) to 5 (= Everyday)
- ‘How often did the teachers ask how you were doing or to tell something about yourself?’ on a scale from 1 (= Never) to 5 (= Everyday)
- ‘How often did the teachers ask what you think of the online subject matter?’ on a scale from 1 (= Never) to 5 (= Everyday)
- ‘How often did your teachers correct your exercises or tasks or did you check them together?’ on a scale from 1 (= Never) to 4 (= Always)
- ‘How often did you miss the online lessons or did you not hand in the online tasks or exercises?’ on a scale from 1 (= Never) to 4 (= Always)
- ‘How often did you learn about the coronavirus at school or about the measures (e.g., keeping distance) and why they are needed?’ on a scale from 1 (= Never) to 4 (= Always)
- ‘How often did you learn something at school about healthy living, e.g. about exercise, healthy eating, or how long you should sit behind a screen?’ on a scale from 1 (= Never) to 4 (= Always)

We first performed an exploratory factor analysis (EFA) with all 9 items. This resulted in a four-factor solution with various cross-loadings. We interpreted factor loadings > 0.3. In this EFA, the following four clusters emerged:

- Factor 1:
  - How often did your teachers correct your exercises or tasks or did you check them together?
  - How often did the teachers ask what you think of the online subject matter?
  - How often did you learn something at school about healthy living, e.g. about exercise, healthy eating, or how long you should sit behind a screen?
  - How often did the teachers ask how you were doing or to tell something about yourself?
- Factor 2:
  - How often did the teachers ask how you were doing or to tell something about yourself?
  - How often did you learn about the coronavirus at school or about the measures (e.g., keeping distance) and why they are needed?
  - What percentage (%) of your teachers gave online tasks, exercises, or lectures to make you learn?
- Factor 3:
  - How often did the teachers ask how you were doing or to tell something about yourself? *(negatively loaded)*
  - What percentage of the subjects matter you received was new?
  - What percentage of your teachers gave online tasks, exercises or lectures to make you learn?
- Factor 4:
  - How often did you receive online tasks, exercises or lectures?
  - How often did you miss the online lessons or did you not hand in the online tasks or exercises?

Since factor 1 and 3 were most related to what we aimed to measure, we reran another EFA while only retaining these 6 items. We again only interpreted factor loadings > 0.3. The EFA indicated two clear factors without any cross-loadings:

- Factor 1:
  - How often did your teachers correct your exercises or tasks or did you check them together?
  - How often did you learn something at school about healthy living, e.g. about exercise, healthy eating, or how long you should sit behind a screen?
  - How often did the teachers ask what you think of the online subject matter?
  - How often did the teachers ask how you were doing or to tell something about yourself?
- Factor 2:
  - What percentage of the subject matter you received was new?
  - What percentage of your teachers gave online tasks, exercises or lectures to make you learn?

Whereas factor 2 seemed more about the amount of learning materials, factor 1 tapped into quality of online instruction. We therefore retained these 4 items for our final scale and ran a confirmatory factor analysis (CFA) with them. This confirmed one underlying factor with factor loadings > 0.6.

**3. Exploratory analyses on psychological adjustment**

We also explored the role of school and home contexts for psychological adjustment during school closure. Life satisfaction (T1, T2, T3) was assessed with five items (e.g., ‘How satisfied are you with your life in general’; Fujita & Diener, 2005; Oishi & Diener, 2001; Phalet et al., 2018) on a scale from 0 (*not satisfied at all*) to 10 (*very satisfied*; α_T1_ = 0.78; α_T2_ = 0.79; α_T3_ = 0.87).

**Main effects**. We did not find any significant main effects of the study variables on psychological adjustment during school closure (CFI = 0.89, RMSEA = 0.03).

**Moderation*.*** We found two significant interactions on life satisfaction (Figure S1). First, a significant interaction between parental education and family support with homework (*B* = -2.01, *SE* = 0.54, β = 0.48, *p* < .001; CFI = 0.75, RMSEA = 0.05) showed that for adolescents with lower educated parents, more family support with homework was associated with *increased* psychological adjustment during school closure (Wald χ^2^ (1) = 5.40, *p* = .020), whereas it was associated with *decreased* psychological adjustment for those with higher educated parents (Wald χ^2^ (1) = 6.88, *p* = .009). Second, we found a significant interaction between subjective SES and teacher rejection (*B* = 0.83, *SE* = 0.41, β = 0.26, *p* = .044; CFI = 0.58, RMSEA = 0.06; Figure S1) so that perceived teacher rejection was associated with decreased psychological adjustment during school closure only among adolescents with lower subjective SES (χ^2^ (1) = 5.92, *p* = .015) but not among those with higher subjective SES (*p* > .10).

**Mediation*.*** Whereas the effects of ethnic origin and subjective SES on the contextual factors were largely the same as in the model for school adjustment (see the main paper; CFI = 0.98, RMSEA = 0.04; Table S1 and Figure S2), we additionally found that adolescents with higher subjective SES reported more teacher support. In turn, there were indications that more teacher support protected psychological adjustment during school closure. Adolescents from higher SES backgrounds might thus show more psychological adjustment during school closure via more teacher support, even though the indirect effect remained non-significant.

In the model examining long-term implications, none of the direct effects of SES, ethnic origin, nor school adjustment at T1 on school adjustment at T3 was significant or improved model fit significantly. We, therefore, left them out of the final model (CFI = 0.98, RMSEA = 0.04). In terms of long-term implications, we found a significant positive stability path (*B* = 1.12, *SE* = 0.26, β = 1.00, *p* < .001), so that changes in life satisfaction during school closure extended until one year after school closure when school reopened again. None of the indirect effects was significant.

Figure S1. *Interactions between home-schooling context and SES indicators predicting psychological adjustment during school closure.*

+


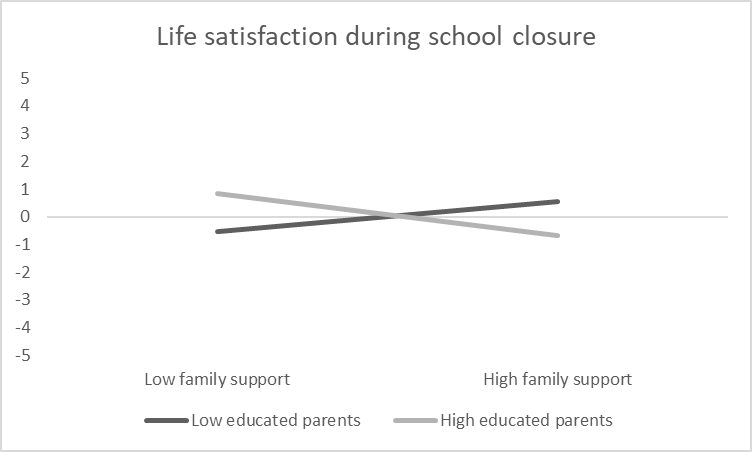


**

**

**

*


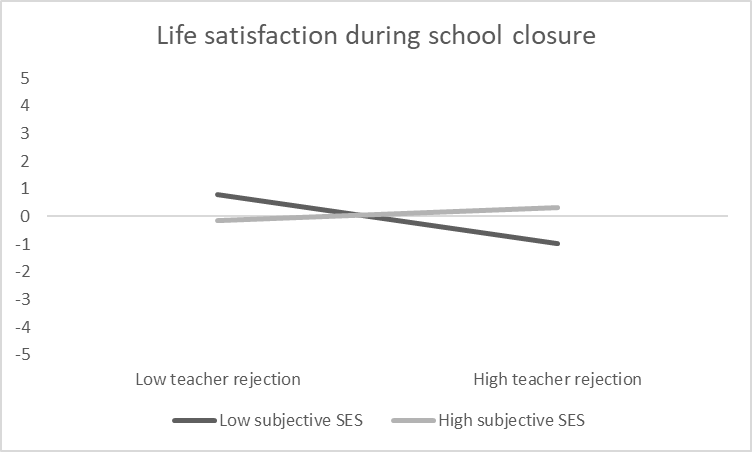


*

+ *p* < .10, * *p* < .05, ** *p* < .01, *** *p* < .001

Table S1. *Mediation model predicting psychological adjustment during school closure.*

|  | Quality of online instruction  B (SE)  β | Family support with homework  B (SE)  β | Resources  B (SE)  β | Teacher support  B (SE)  β | Teacher rejection  B (SE)  β | Psychological adjustment  B (SE)  β |
| --- | --- | --- | --- | --- | --- | --- |
| Psychological adjustment at T1 | -- | -- | -- | -- | -- | 0.63 (0.15)***  0.54 |
| Majority (vs. minority) | -0.13 (0.05)**  -0.33 | 0.46 (0.16)**  0.35 | 0.49 (0.25)*  0.25 | 0.14 (0.14)  0.12 | -0.32 (0.10)**  -0.40 | -0.65 (0.40)  -0.19 |
| Subjective SES | 0.00 (0.02)  0.03 | 0.10 (0.05)*  0.27 | 0.16 (0.08)+  0.29 | 0.10 (0.05)*  0.28 | -0.03 (0.03)  -0.15 | -0.06 (0.13)  -0.06 |
| Higher educated parents (vs. lower) | -0.04 (0.04)  -0.10 | 0.14 (0.15)  0.11 | 0.27 (0.25)  0.14 | 0.06 (0.15)  0.05 | 0.02 (0.09)  0.03 | -0.09 (0.34)  -0.03 |
| Quality of online instruction | -- | -- | -- | -- | -- | -0.36 (1.00)  -0.04 |
| Family support with homework | -- | -- | -- | -- | -- | 0.05 (0.33)  0.02 |
| Resources | -- | -- | -- | -- | -- | 0.26 (0.21)  0.15 |
| Teacher support | -- | -- | -- | -- | -- | 0.56 (0.32)+  0.21 |
| Teacher rejection | -- | -- | -- | -- | -- | -0.65 (0.47)  -0.16 |

*Note.* The mediation model was ran in one model, whereby quality of online instruction, family support with homework, resources, teacher support, and teacher rejection served as mediators, and psychological adjustment at T2 as DV. Effects were standardized using STDYX standardization.

+ *p* < .10, * *p* < .05, ** *p* < .01, *** *p* < .001


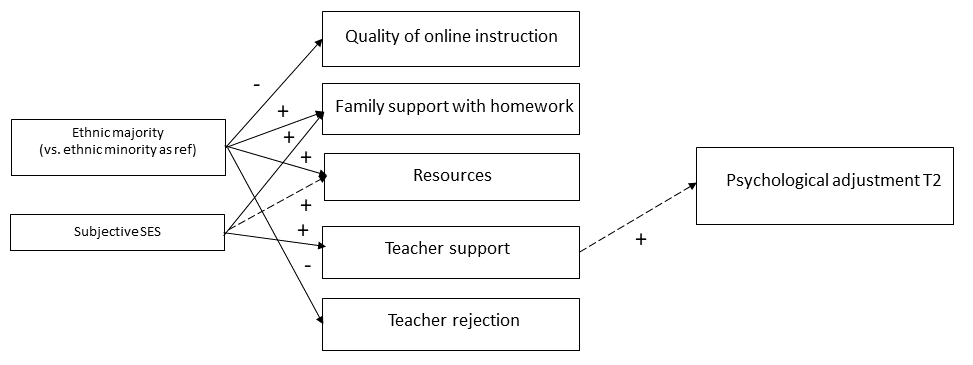
Figure 4. *Mediation model predicting psychological adjustment during school closure*

*Note.* Only significant paths are displayed. Dashed lines indicate .05 < *p* < .10. Parental education was also included in the model but did not show any significant effects and is therefore left out of the figure.

**4. Measurement invariance across time**

We assessed measurement invariance across time. We compared a fully constrained model (i.e., scalar invariance; factor loadings and item intercepts constraint to be equal at the three different timepoints) to a model with freely estimated item intercepts and constrained factor loadings (i.e., metric invariance), and to a freely estimated model (i.e., configural invariance; freely estimated factor loadings and intercepts). As the chi-square test is sensitive to sample size (Cheung & Rensvold, 2002, Structural Equation Modeling), other established criteria were additionally used to evaluate difference in model fit. Specifically, an increase of 0.010 or larger in CFI supplemented by a decrease of 0.015 in RMSEA or larger or by a decrease of 0.030 in SRMR or larger would indicate significant differences in model fit (Chen, 2007, Structural Equation Modeling). Looking at the combined criteria, results (Table S2) showed that all school adjustment outcomes demonstrated scalar invariance across time. Both factor loadings and item intercepts were therefore equal across time.

Table S2*: Measurement invariance across time*

|  | CFI (ΔCFI) | RMSEA (ΔRMSEA) | SRMR (ΔSRMR) | χ^2^ (df) | Correction factor | Δχ^2^ (df) |
| --- | --- | --- | --- | --- | --- | --- |
| School belonging |  |  |  |  |  |  |
| Scalar | 0.773 (--) | 0.109 (--) | 0.102 (--) | 155.45 (63) | 0.94 | -- |
| Metric | 0.784 (0.011) | 0.112 (0.003) | 0.102 (0.000) | 144.86 (57) | 0.95 | 10.04 (6) |
| Configural | 0.786 (0.002) | 0.118 (0.006) | 0.094 (-0.008) | 138.32 (51) | 0.96 | 5.16 (6) |
| School engagement |  |  |  |  |  |  |
| Scalar | 0.534 (--) | 0.091 (--) | 0.143 (--) | 713.05 (353) | 0.99 | -- |
| Metric | 0.547 (0.013) | 0.092 (0.001) | 0.142 (-0.001) | 687.11 (337) | 1.00 | 24.37 (16)+ |
| Configural | 0.546 (-0.001) | 0.094 (0.002) | 0.137 (-0.005) | 672.15 (321) | 0.99 | 15.75 (16) |
| Academic self-esteem |  |  |  |  |  |  |
| Scalar | 0.781 (--) | 0.074 (--) | 0.102 (--) | 105.21 (63) | 0.96 | -- |
| Metric | 0.864 (0.083) | 0.061 (-0.013) | 0.090 (-0.012) | 83.27 (57) | 0.98 | 25.03 (6)*** |
| Configural | 0.849 (-0.015) | 0.068 (0.007) | 0.091 (0.001) | 80.09 (51) | 0.98 | 3.06 (6) |

*Note.* Chi-square difference test refer to the robust Santorra Bentler chi-square difference test.

+ *p* < .10, * *p* < .05, ** *p* < .01, *** *p* < .001

**5. Lagged regression models**

Below we report the lagged regression models in which we predicted school adjustment during school closure (T2; Table S2) and one year after school closure (T3; Table S3) from adolescents’ ethnic and SES backgrounds, while controlling for prior levels of adjustment.

Table S3. *Lagged regression model predicting school adjustment at T2*

|  | Belonging T2 | Engagement T2 | Academic self-esteem T2 |
| --- | --- | --- | --- |
|  | *B (SE)*  β | *B (SE)*  β | *B (SE)*  β |
| Control variables |  |  |  |
| Outcome T1 | 0.42 (0.11)***  0.45 | 0.61 (0.12) ***  0.55 | 0.40 (0.12)**  0.38 |
| Girls (vs. boys) | -0.24 (0.14)+  -0.17 | -0.01 (0.11)  -0.01 | -0.08 (0.15)  -0.06 |
| Vocational (vs. academic) | -0.14 (0.16)  -0.10 | 0.22 (0.13)  0.18 | 0.05 (0.17)  0.04 |
| Age | 0.13 (0.09)  0.17 | 0.03 (0.08)  0.04 | -0.09 (0.11)  -0.12 |
| Main variables |  |  |  |
| Majority (vs. minority) | 0.40 (0.16)*  0.28 | 0.32 (0.13)*  0.27 | 0.41 (0.16)*  0.29 |
| Subjective SES | 0.03 (0.05)  0.08 | 0.02 (0.04)  0.06 | 0.05 (0.05)  0.13 |

*Note.* Analyses were run separately per DV. Effects were standardized using STDYX standardization.

+ *p* < .10, * *p* < .05, ** *p* < .01, *** *p* < .001

Table S4. *Lagged regression model predicting school adjustment at T3*

|  | Belonging T3 | Engagement T3 | Academic self-esteem T3 |
| --- | --- | --- | --- |
|  | *B (SE)*  β | *B (SE)*  β | *B (SE)*  β |
| Control variables |  |  |  |
| Outcome T2 | 0.85 (0.11)***  0.77 | 0.58 (0.10)***  0.59 | 0.33 (0.15)*  0.31 |
| Girls (vs. boys) | 0.07 (0.16)  0.04 | -0.12 (0.12)  -0.10 | -0.13 (0.16)  -0.09 |
| Vocational (vs. academic) | 0.06 (0.19)  0.04 | 0.02 (0.15)  0.02 | -0.28 (0.21)  -0.18 |
| Age | -0.09 (0.12)  -0.11 | -0.08 (0.09)  -0.13 | -0.24 (0.14)+  -0.28 |
| Main variables |  |  |  |
| Majority (vs. minority) | 0.00 (0.18)  0.00 | 0.06 (0.14)  0.06 | -0.20 (0.19)  -0.13 |
| Subjective SES | -0.12 (0.07)+  -0.26 | 0.05 (0.05)  0.14 | -0.02 (0.08)  -0.06 |

*Note.* Analyses were run separately per DV. Effects were standardized using STDYX standardization.

+ *p* < .10, * *p* < .05, ** *p* < .01, *** *p* < .001
